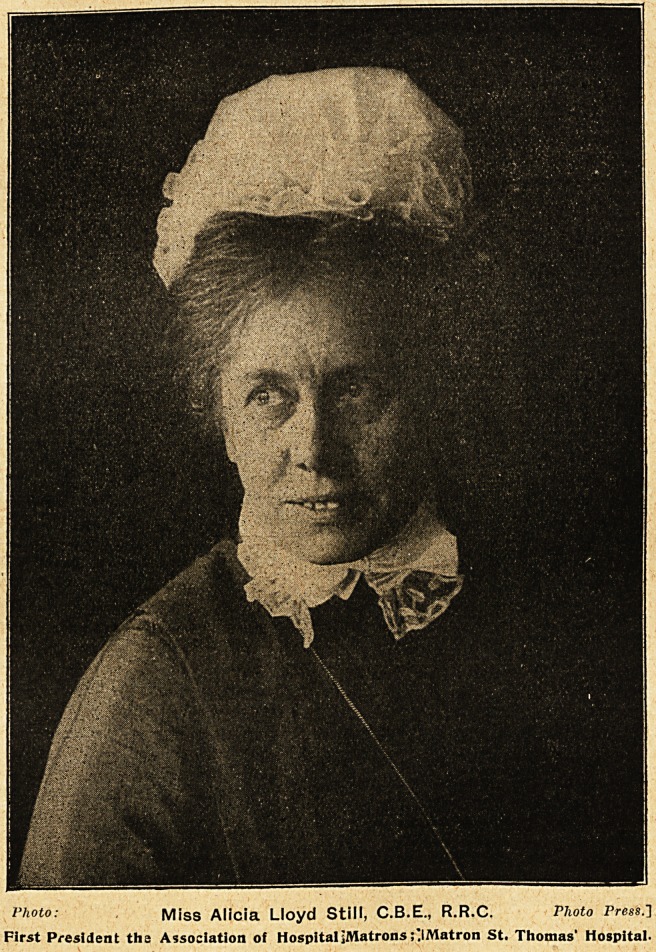# Representative Inaugural Meeting at St. Thomas's Hospital

**Published:** 1919-05-17

**Authors:** 


### NEW ASSOCIATION OF MATRONS.

### Representative Inaugural Meeting at St. Thomas's Hospital.

An association has been formed for England and Wales of trained nurses who hold, or have held, the position of matron or superintendent of hospitals or institutions concerned in the training of nurses and the care of the sick. On the initiative of a few leading matrons steps were taken which resulted on Thursday in a meeting at St. Thomas's Hospital, London, of heads of the nursing profession. The representative character of the gathering is demonstrated by the following list of the names of those who attended :—

C. Alcock, Matron, Royal Hospital, Portsmouth; H. A. Alsop, Matron, Kensington Infirmary; J. M. T. Babtie, Matron, St. George's Hospital, Hyde Park Corner; A. B. Baillie, Matron, Royal Infirmary, Bristol; L. Barrow, Matron, Northern Hospital, Manchester; E. C. Barton, Matron, Chelsea Infirmary; M. Barwell, Matron, General Hospital, Great Yarmouth; I. C. Bennett, Matron, Metropolitan Hospital, N.E.; E. Berry, Matron, Surbiton Hospital, Surrey; M. Bird, Matron, Croydon General Hospital; A. Blomfield, Matron, Queen Charlotte's Hospital; E. Booth, Matron, City of Westminster Infirmary, Fulham Road; S. A. Brown, Matron, General Military Hospital, Colchester; Adeline Cable, Matron, Salisbury General Infirmary; F. Cann, Matron, Norwich; A. Clack, Matron, Cuddington Hospital, Banstead; L. S. Clark, Matron, Whipps Cross Infirmary, Leytonstone; M. K. Coggins, Matron, Royal Infirmary, Wigan; A. M. Coulton, Matron, East London Hospital for Children, Shadwell; R. Cox Davies, Matron, Royal Free Hospital; E. M. Crawford, Matron, Westminster Training-School, Queen Anne's Gate; C. E. Crookenden, Matron, Addenbrooke's Hospital, Cambridge; L. C. Dalton, Matron, City of London Chest Hospital; V. Daunt, late Matron, London Homœopathic Hospital; T. Davey, Sheffield Royal Hospital; A. E. Densham, Matron, General Hospital, Bristol; E. Dodds, Matron, Bethnal Green Military Hospital; E. A. Eddison, Matron, London Homœopathic Hospital; A. Edgar, Matron, General Hospital, Ramsgate; E. M. Edwards, Matron, Maternity Hospital, Leeds; D. Finch, Matron, University College Hospital; A. C. Gibson, late Matron, Birmingham Poor-Law Infirmary; M. Girdlestone, late Matron, Crumpsall Infirmary; E. Gomm, Matron, St. Peter's Hospital, Henrietta Street, W.C. 2; E. Goodall, Sister-in-Charge, Red Cross Hospital, Streatham, S.W.; A. Grasett, Matron, Infants' Hospital, Vincent Square; Mrs. E. Gray, late Matron, St. George's Hospital, Hyde Park Corner; R. Gregory, Matron, Hampstead General Hospital; C. Hall, Matron, St. Mark's Hospital, City Road; E. Hall, Matron, Seamen's Hospital Greenwich; L. Halliday, Matron, Royal Waterloo Hospital; J. C. Hancock, Matron, Evelina Hospital for Children; H. Hannath, Matron, General Hospital, Wolverhampton; E. B. Harradine, late Matron Royal South Hants Hospital, Southampton; M. Hogg, Matron Guy's Hospital, S.E. 1; S. Hutchinson, Matron, Coventry and Warwickshire Hospital, Coventry; E. S. Innes, Matron, General Infirmary, Leeds; K. Jackson, Matron, St. Mary's Hospital, Paddington; F. Knowles, Matron, 4th London General Hospital; F. C. Lorrimar, Matron, Sobraon Military Hospital, Colchester; A. Lloyd Still, Matron, St. Thomas's Hospital, S.E. 1; A. McIntosh, Matron, St. Bartholomew's Hospital; I. MacMaster, Matron, North Staffordshire Infirmary, Stoke-on-Trent; E. A. Millar, Matron, 1st London General Hospital: A. M. T. Millington, retired Matron, Hong Kong Government Civil Hospital; F. E. Mason, Matron, Royal United Hospital, Bath; M. G. Montgomery, Matron, Middlesex Hospital; H. Morgan, Matron, General Hospital, Darlington; E. W. Mowat, Matron, Whitechapel Infirmary; E. M. Musson, Matron, General Hospital, Birmingham; M. L.

*Photo:* Miss Alicia Lloyd Still, C.B.E., R.R.C. *Photo Press.*]

**First President the Association of Hospital Matrons; Matron St. Thomas' Hospital.**

**New Association of Matrons**—(*continued*).

Pollett, Matron, Royal London Ophthalmic Hospital, City Road; F. Redl, Brompton Hospital for Diseases of the Chest, Fulham Road; M. S. Riddell, T.F.N.S., Matron, War Office, 80 Pall Mall; A. Row, late Lady Superintendent, East London Hospital for Children; M. Scovell, Matron, General Hospital, Swansea; J. A. Sheldon, Superintendent, Guy's Trained Nurses' Institute; I. A. Sheppard, Matron, County Hospital, Lincoln; B. C. Sherratt, Matron, Cancer Hospital, Fulham Road; E. Smith, Matron, The Infirmary, Watford; E. Smith, Matron, Westminster Hospital; W. C. Smeeton, Matron, Royal Infirmary, Sheffield; A. M. Spackman, Matron, National Hospital, Queen Square; M. C. Sparshott, Lady Superintendent, Royal Infirmary, Manchester; F. S. Spittle, Matron, St. Pancras Infirmary, Highgate; M. E. Sutcliffe, Matron, Derbyshire Royal Infirmary; M. Stanford, Matron, Her Majesty's Hospital, Stepney Causeway, E. 1; E. Carpenter Turner, Matron, Royal Hampshire County Hospital, Winchester; C. E. Todd, Matron, St. James's Infirmary; E. M. Vezey, late Matron, Salisbury General Infirmary; L. M. Wainwright, Matron, Archer House, Ramsgate; E. L. Walker, Matron, British Home and Hospital for Incurables, Streatham; F. C. Wallen, Matron, St. Mary, Islington, Infirmary, Highgate Hill; I. Watson, Matron, Victoria Hospital, Chelsea; A. Watt, Matron, Radcliffe Hospital, Oxford; E. West, Matron, Chelsea Hospital for Women; M. Whiffin, Matron, 2nd Northern General Hospital, Leeds; M. E. Windemer, Matron, Freemason War Hospital, Fulham Road; E. A. Wynne, Matron, Royal Berkshire Hospital, Reading.

### Not a Rival Association.

Miss Lloyd Still, Matron, St. Thomas's Hospital, Superintendent of the Nightingale Training-School, who was voted to the chair, said she was pleased to greet so many of her colleagues and fellow-workers at that meeting, which seemed to promise great things for the future. She was anxious to make two statements: First, that this is not to be a rival association to any existing on the same lines. It was not formed with that idea, and she trusted it would never follow such a policy. Secondly, she thought a vote of thanks is due to the College of Nursing for having made it possible that such a gathering could be formulated. The College policy had ever been to work for unity, and that, she thought, was proved by the fact that the heads of the profession have shown such sympathy with the formation of the association now proposed, and that they were able to meet in such numbers with a united aim. (Applause.)

### The History of the Movement.

Miss Cox-Davies, Royal Free Hospital, one of the Hon. Provisional Secretaries, gave a brief history of the movement. She said that on April 13 some of them in London talked the matter over and came to the conclusion that it would be advantageous to have an association like that now under consideration, to enable leaders in the nursing world to meet and discuss problems which concerned the profession. They communicated with some provincial matrons, and later a letter, to which six names were appended, was sent to about forty matrons or superintendents. Those who signed this letter were Miss Cummins, Liverpool Royal Infirmary; Miss Gill, Edinburgh; Miss Cox-Davies, Royal Free Hospital; Miss Finch, University College Hospital; Miss McIntosh, St. Bartholomew's Hospital; Miss Sparshott, Royal Infirmary, Manchester; and Miss Lloyd Still, St. Thomas's Hospital. The forty matrons selected, it was thought, would give a fairly representative guidance as to whether the movement would receive support. All were asked to reply by telegram. By the 15th all, with one exception, had replied expressing warm sympathy with the project. In order that no time might be lost in taking the necessary steps a meeting at St. Thomas's Hospital was called by telephone for the afternoon of April 15, when fifteen ladies were present. At this meeting it was decided unanimously to form the association, and the following were elected a Provisional Committee to draw up the draft constitution and arrange for a general meeting:—Miss Gibson, Miss Cox-Davies, Miss Lloyd Still, Miss Finch, Miss Blomfield (Queen Charlotte's Hospital), Miss Barton (Chelsea Infirmary), Miss Montgomery (Middlesex Hospital), Miss Musson (General Hospital, Birmingham), Miss Vincent, and Miss Hogg (Guy's Hospital). The secretarial duties were undertaken by Miss Lloyd Still and the speaker. This committee met three times, under the chairmanship of Miss Gibson, and drew up the proposals placed before the meeting that day. Notices of the meeting had been as widely circulated as time would allow. Replies had been received. Ninety accepted the invitation, thirty-seven were not able to be present, nine paid their subscription—(laughter)—and three were non-committal. The word "rival" had been used in a section of the nursing Press in connection with this association. She hoped, however, that the word would never be justified, and that controversy would be unknown. (Hear, hear.) She was pleased to have the opportunity of endorsing what Miss Lloyd Still had said about the College of Nursing. They owed the College a debt of gratitude for the helpful work it had accomplished for the profession, as well as the sympathy it had displayed towards the present movement. (Applause.)

### The Association Agreed Upon.

Miss Finch then proposed confirmation of the following resolution passed at the meeting on April 15: "That an association be formed of trained nurses who hold, or have held, the position of matron or superintendent of hospitals and institutions concerned in the training of nurses and the care of the sick," and said she was convinced that an association of the kind propounded would be of great benefit to the profession as a whole.

Miss Gibson, who seconded, said it was a great joy to her to see such a meeting, which a few years ago would have been impossible. The new feeling which had grown up was entirely due to the influence of the College of Nursing. The association now proposed would be a great influence for good in the nursing world.

In the course of the discussion which followed it was asked why Scotland and Ireland were not included in the scheme.

The Chairman explained that similar associations were already in existence in Scotland and Ireland, and it was hoped that if an English association was formed all three would in the not distant future be affiliated.

The resolution was carried.

### The Constitution.

Miss Hogg then proposed the following scheme:—

The Association shall be called "The Association of Hospital Matrons."

#### Objects.

1. To enable members to take counsel together on matters affecting their profession.
2. To consider, and if necessary take action, upon legislative proposals calculated to affect the interests of the nursing profession.
3. To maintain the honour and further the interests of the nursing profession.

#### Constitution.

1. The Association shall be formed of trained nurses who hold, or have held, the position of matron or superin-

**New Association of Matrons—(*continued*).**

tendent of hospitals or institutions concerned in the training of nurses and the care of the sick.

2. There shall be a President, Hon. Secretary, and an Hon. Treasurer, with an Executive Committee of fifteen members, the hon. officers to be ex-officio members of Committee. Six shall form a quorum. One-third of the Committee shall retire each year. Hon. officers shall hold office for three years.

3. The Executive Committee shall meet monthly. The Association shall meet quarterly and at such other times as may be required on the summons of the President or on a requisition in writing signed by six members of the Association, stating the business to be considered, and addressed to the Hon. Secretary at least ten days before the proposed meeting.

4. For the purpose of avoiding the possibility of plural voting, the carefully considered opinion of the Association on matters of professional importance shall be expressed as from the Association, and not by individual members.

5. The annual subscription of 5s. shall be payable on March 1.

Miss Innes (Leeds Royal Infirmary) seconded.

Miss Musson suggested that the language of the first clause of the Constitution might lead to the impression that only matrons of training-schools were eligible. And the small hospitals did so want help.

The Chairman said they had tried to make it clear that the matrons of all hospitals, small as well as large, were eligible for membership. She would like to add that the Constitution had been modelled on that of the Scottish Association; it was practically the same.

Miss Musson further suggested that the first part of Clause 4 was hazy; and, after discussion, it was decided, on the motion of Miss Sparshott, seconded by Miss Finch, to omit the words "For the purpose of avoiding the possibility of plural voting." With this exception the Constitution was adopted in the form given above.

Election of President and Hon. Officers.

Miss Cox-Davies then took the chair, and proposed that Miss Lloyd Still be the first President of the Association. In this movement, she said, Miss Lloyd Still has been an inspiration and a great help. There could be no one more fitting as President than the matron of St. Thomas's Hospital, apart from the fact that Miss Lloyd Still was known to them all and greatly respected for her work.

Miss Musson seconded, and said she hoped the Association over which Miss Lloyd Still would preside would soon grow into a large one.

The resolution having been carried,

The President was warmly greeted on resuming her seat. She thanked them for having paid her the honour of being the first President of an Association which bids fair to have many years of work and responsibility before it. It would be her earnest endeavour to help in every possible way the work they had set themselves to do. Unity was strength, and in that unity they would accomplish great things for their profession, and uphold its traditions and ideals, which none but those who have gone through the training of a nurse could grasp or understand. The nursing world was passing through a critical period. Reforms had taken place during the past few years, but much more remained to be done for the good of our nurses and training-schools. Surely the responsibility of those reforms could best be undertaken by those whose lives had been given up to the profession. (Applause.)

On the motion of Miss Montgomery, seconded by Miss Barton, Miss Finch was elected Hon. Treasurer; and on the proposition of Miss Gibson, seconded by Miss McIntosh, Miss Cox-Davies was chosen Hon. Secretary.

Miss Crookender (Cambridge) proposed, Miss Hall (Greenwich) seconded, and it was agreed that the following be the Executive Committee: Miss Baillie (Bristol Royal Infirmary), Miss Barton (Chelsea Infirmary), Miss Blomfield (Queen Charlotte's Hospital), Miss Coulton (Children's Hospital, Shadwell), Miss Cummins (Liverpool Royal Infirmary), Miss Gibson, Miss Hogg (Guy's Hospital), Miss Masters (Leicester Poor-Law Infirmary), Miss McIntosh (St. Bartholomew's Hospital), Miss Monk (The London Hospital), Miss Montgomery (The Middlesex Hospital), Miss Musson (General Hospital, Birmingham), Miss Peterkin (Queen's Jubilee Nurses), Miss Redl (Hospital for Consumption, Brompton), Miss Sparshott (Royal Infirmary, Manchester).

A vote of thanks to the Treasurer and the Governors of St. Thomas's Hospital closed the proceedings.

## Figures and Tables

**Figure f1:**